# A Genome-wide View of Transcriptome Dynamics During Early Spike Development in Bread Wheat

**DOI:** 10.1038/s41598-018-33718-y

**Published:** 2018-10-18

**Authors:** Yongpeng Li, Xing Fu, Meicheng Zhao, Wei Zhang, Bo Li, Diaoguo An, Junming Li, Aimin Zhang, Renyi Liu, Xigang Liu

**Affiliations:** 1 State Key Laboratory of Plant Cell and Chromosome Engineering, Center for Agricultural Resources Research, Institute of Genetics and Developmental Biology, Chinese Academy of Sciences, Shijiazhuang, 050021 China; 2 Shanghai Center for Plant Stress Biology, Chinese Academy of Sciences, Shanghai, 201602 China; 3 College of Life Sciences, University of Chinese Academy of Sciences, 19A Yuquan Road, Beijing, 100049 China; 4 Center for Agricultural Resources Research, Institute of Genetics and Developmental Biology, Chinese Academy of Sciences, Shijiazhuang, 050021 China; 5 State Key Laboratory of Plant Cell and Chromosome Engineering, Institute of Genetics and Developmental Biology, Chinese Academy of Sciences, Beijing, 100101 China; 6 Center for Agroforestry Mega Data Science and FAFU-UCR Joint Center for Horticultural Biology and Metabolomics, Haixia Institute of Science and Technology, Fujian Agriculture and Forestry University, Fuzhou, 350002 China

## Abstract

Wheat spike development is a coordinated process of cell proliferation and differentiation with distinctive phases and architecture changes. However, the dynamic alteration of gene expression in this process remains enigmatic. Here, we characterized and dissected bread wheat spike into six developmental stages, and used genome-wide gene expression profiling, to investigate the underlying regulatory mechanisms. High gene expression correlations between any two given stages indicated that wheat early spike development is controlled by a small subset of genes. Throughout, auxin signaling increased, while cytokinin signaling decreased. Besides, many genes associated with stress responses highly expressed during the double ridge stage. Among the differentially expressed genes (DEGs), were identified 375 transcription factor (TF) genes, of which some homologs in rice or *Arabidopsis* are proposed to function in meristem maintenance, flowering time, meristem initiation or transition, floral organ development or response to stress. Gene expression profiling demonstrated that these genes had either similar or distinct expression pattern in wheat. Several genes regulating spike development were expressed in the early spike, of which *Earliness per se 3* (*Eps-3*) was found might function in the initiation of spikelet meristem. Our study helps uncover important genes associated with apical meristem morphology and development in wheat.

## Introduction

Several cereal crops are important staple foods that feed populations worldwide and are therefore critical for global food security^1^. Wheat (*Triticum aestivum*) is one of the most important staple crops and provides about 20% of the calories to the human population. Crop yield is a multifactorial trait and is mainly determined by grain number (GN) and mean grain weight. Final GN results from the development of the spike, which includes spikelet and floret^2^. Although the combined use of functional genomics, bioinformatics and genetic resources has led to the identification of numerous key regulators in spike development in rice and maize, the underlying regulatory mechanisms have yet to be fully characterized^3–5^. Fewer advances have been made towards understanding spike development in wheat due to its polyploidy, large genome size (17 Gb) and low transformation efficiency.

Plant meristems are responsible for the generation of all plant tissues and organs. In the model eudicot *Arabidopsis*, indeterminate inflorescence meristems (IMs) generate floral meristems (FMs), which subsequently produce all the floral organ primordia and eventually result in a simple raceme-type inflorescence. In contrast, monocot crops, such as rice, maize and wheat, develop panicle-type inflorescences, in which the main IMs terminate or abort after generating a series of branch meristems (BMs) that give rise to secondary branch meristems (SBMs) or spikelet meristems (SMs), followed by the initiation of FMs^5^. Spike development comprises a set of sequential developmental events featured by transitions from the early IMs to BMs, then to SBMs/SMs and the final FMs. Numerous genes contributing to this process have conserved functions between eudicots and monocots^5–7^. The spikelet is the basal unit of crop inflorescences and is composed of leaf-like bracts known as glumes and one to several florets therein. While some genera, such as *Oryza*, *Sorghum* and *Setaria*, have long, branched inflorescences, wheat and barley develop compact inflorescences without secondary branches and with the spikelets attached to the main axis. The morphological diversity of inflorescences suggests that different genes or homologous genes with divergent functions may be involved in the spike development of different crop species. Based on developmental anatomy and morphology, early spike development in wheat can be separated into six distinct phases: the vegetative, elongation, single ridge, double ridge, glume differentiation and floret differentiation stages^8^. In *Arabidopsis* and rice, inflorescence development is a cascade of developmental events controlled by many genes with distinct spatio-temporal expression patterns and phytohormones such as auxin and cytokinin^5,9^. In contrast, the key regulators and genetic networks involved in wheat early spike development are still largely unknown.

Bread wheat is an allohexaploid (2n = 6× = 42, AABBDD) formed by the hybridization of allotetraploid *Triticum turgidum* (2n = 4× = 28, AABB) and the diploid wild goat grass *Aegilops tauschii* (2n = 2× = 14, DD), followed by chromosome doubling, resulting in a large genome size (17 Gb, 40 times the size of the rice genome) and a large proportion of repetitive DNA (>80%)^10–12^. As a consequence, hexaploid wheat has three distinct but closely related subgenomes (A, B and D), which contain homoeologous genes that usually have over 95% sequence identity and are distributed in a collinear manner^13^. The large genome size and the high sequence conservation among homoeologous genes have hampered functional genomic studies in bread wheat. Recently the reference sequences of natural hexaploid wheat (Chinese Spring) and the A and D donor genomes have been generated^10,14,15^. These genomic resources not only permit comparative and evolutionary analyses with closely related species such as rice and *Brachypodium*^16^, but also make it much easier to dissect the molecular mechanisms underlying important developmental processes in wheat.

Early studies of high throughput gene expression profiling in wheat relied on DNA microarray technologies with probes corresponding to unigenes from the publicly available expressed sequence tag (EST) resources^17,18^. This method was relatively low throughput and high cost and also suffered from deviation in the quantitative analysis^19^. The next-generation sequencing (NGS) technology overcomes some of the shortcomings of microarrays and has become the method of choice for transcriptome profiling in recent years. Although NGS has been used more widely in other plant species such as *Arabidopsis* and rice, its application for transcriptome analysis in wheat is still limited. Nevertheless, several studies have used NGS to investigate the mRNA and small RNA transcriptomes, gene expression additivity, and single nucleotide polymorphisms (SNPs) in natural hexaploid wheat and other wheat varieties^20–22^. Here, we performed transcriptome profiling of stage-specific spikes from bread wheat, followed by comprehensive analysis, to uncover the dynamic alteration of gene expression. By providing a broad view of dynamic genome-wide gene expression in early wheat spike development, our results help uncover important genes associated with apical meristem morphology and development in wheat.

## Results

### Stage-specific wheat spikes for RNA-seq analysis

**Figure 1:**
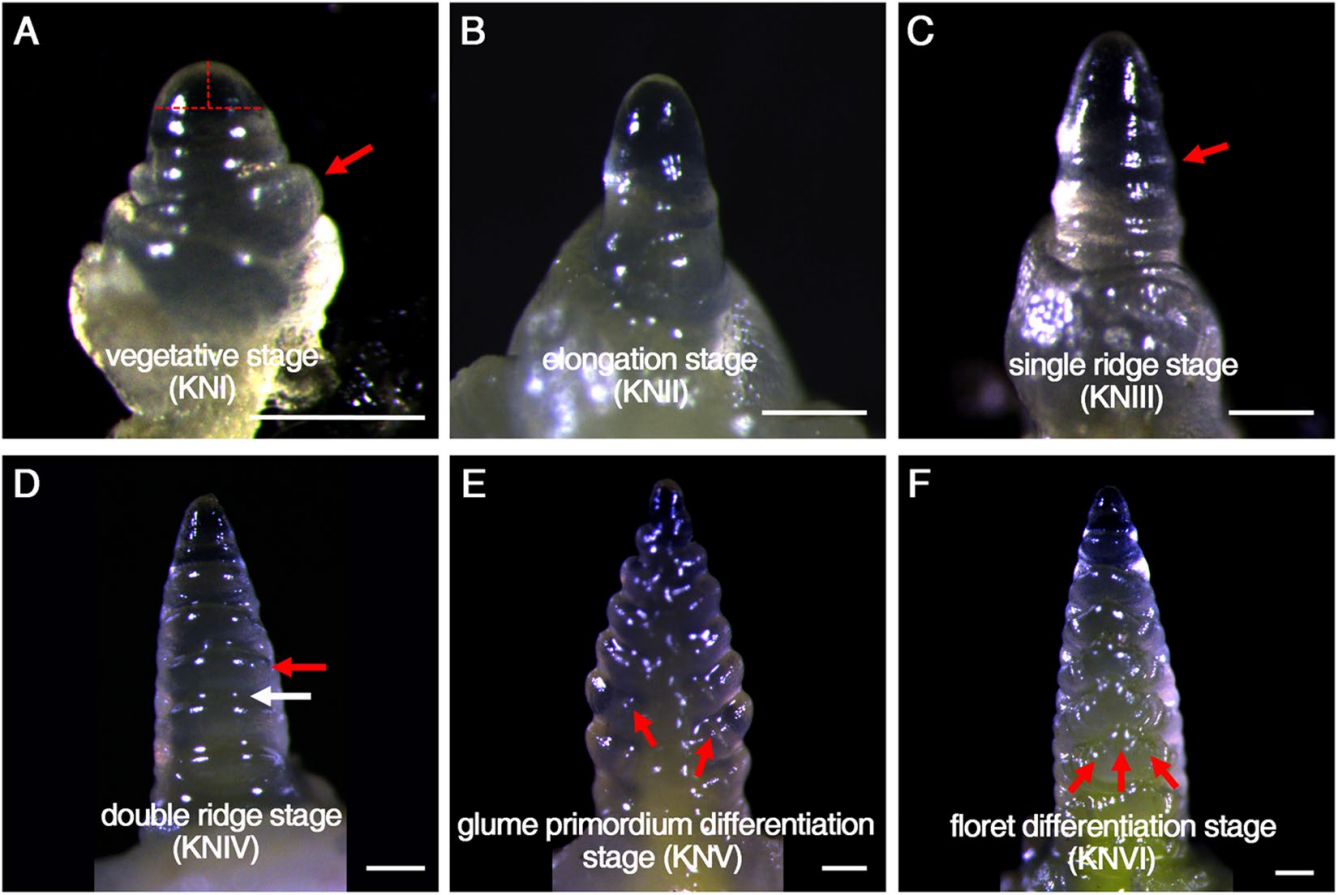
The bread wheat spikes in six developmental stages that were used for gene expression analyses. All samples shown are from Kenong9204 (KN9204). (**A**) Vegetative stage (KNI). Leaf primordial is indicated by a red arrow. Length and width of meristem are marked by dot lines. (**B**) Elongation stage. (**C**) Single ridge stage. Bracteal primordial is indicated by a red arrow. (**D**) Double ridge stage. Bracteal primordial (lower ridge) is indicated by a white arrow. Spikelet meristem (upper ridge) is indicated by a red arrow. (**E**,**F**) glume primordium differentiation stage (**E**) and floret differentiation stage (**F**). Floral meristems are indicated by red arrows. Bar: 200 µm.

Wheat cultivar Kenong 9204 (KN9204) was used in this study because it is a winter wheat with normal grain numbers compared to other bread wheat varieties and is widely used in the wheat research community. The genetic map of KN9204 and several genetic populations derived from KN9204 are well characterized, making it ideal for genetic analysis^23–25^. To minimize the impact of various environmental factors, such as light and temperature variation and salt or drought stress, on wheat spike development, we cultivated wheat in growth rooms after 40 days of cold treatment at 4 °C after seed germination, as previously described^26^. Stage-specific wheat spikes were dissected under a stereomicroscope based on anatomic and morphological features according to established procedures (Fig. 1)^8^. At the vegetative stage (denoted KNI in Fig. 1) that lasted about two weeks, the short and hemispherical growing point, corresponding to a meristem with a low length-to-width ratio, was responsible for the generation of leaf primordia (Fig. 1A). Then, the growing point elongated in preparation for spike development in a phase known as the elongation stage (KNII) (Fig. 1B) when the fifth leaf has just come out 1 week after KNI stage. Then, the single ridge stage (KNIII) lasted 4–7 days, when the seedlings showed five leaves and the bracteal initials instead of leaf primordia were produced, indicating the transition from vegetative stage to reproductive stage (Fig. 1C). The double ridge stage (KNIV) was considered the initial reproductive stage that lasted only 3–5 days when seedlings showed five leaves and one heart or six leaves morphologically. While the lower ridge (Fig. 1D; indicated by the white arrow) of the pair would eventually degenerate, the upper ridge (Fig. 1D; indicated by the red arrow), corresponding to the spikelet meristem, would elongate and initiate spikelet development. The glume primordium differentiation stage (KNV) was relatively shorter than the other stages (2–3 days after KNIV stage), but marked the transition from the spikelet meristem to the floral meristem (Fig. 1E). At the floret differentiation stage (KNVI) when seedlings have 6–7 leaves, floral meristems were produced and several floret primodia were generated for each spikelet (Fig. 1F). It was worth noting that we collected the tissues before floral organogenesis and, therefore, the samples consisted of specifically meristematic cells from SAMs, IMs, SM and FMs as well as a few differentiated cells that have little impact on the consequent data analysis.

### Global analysis of gene expression in wheat spike development

**Figure 2:**
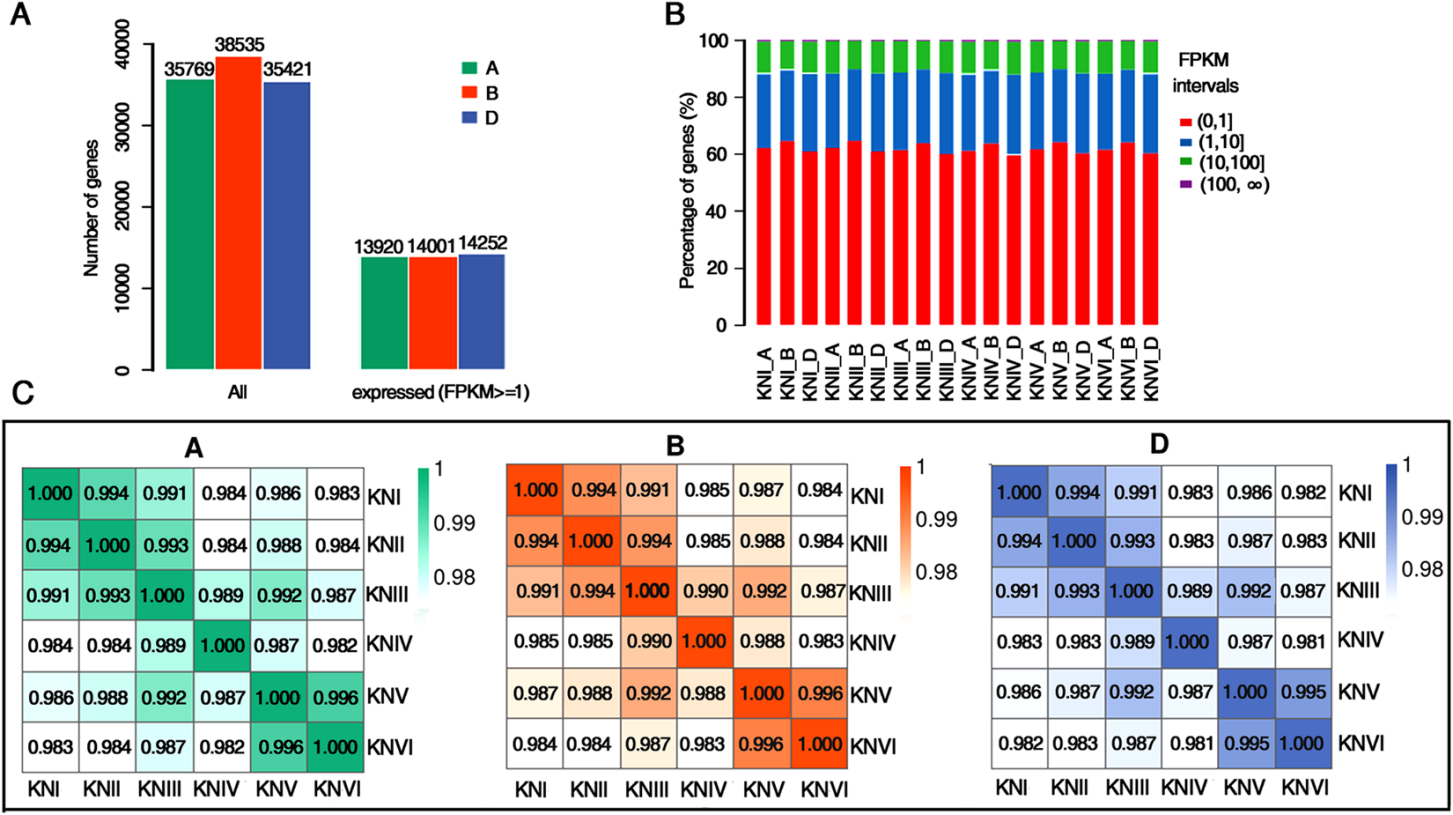
Overview of the wheat spike gene expression profiles at six developmental stages. (**A**) Total number of annotated genes in the A, B and D subgenomes (left) and number of genes that were expressed in at least one developmental stage (right, FPKM ≥ 1). (**B**) The proportion of genes with the indicated expression strength at each developmental stage. The strength of expression is divided into four categories according to the normalized expression level (fragments per kilobase of exon model per million mapped reads, FPKM). (**C**) The Pearson correlation coefficients (PCCs) of gene expression (FPKM) between stages. The PCCs of expressed genes from the A, B and D subgenomes were calculated separately.

To obtain the global gene expression patterns during wheat spike development, we collected samples with two biological replicates at six developmental stages and performed RNA-seq using paired-end libraries with ~200 bp inserts (PE125) and obtained approximately 30 million read pairs for each sample. We followed our well-established bioinformatics workflow for the data analysis (Supplementary Fig. S1). The latest bread wheat genome (TGACv1) sequence and annotation data were downloaded from Ensembl Plants database and used as the reference for the data analysis. The RNA-seq read pair mappings were classified into six alignment scenarios as previously described^27^ with slight modifications (Supplementary Fig. S2). Over 80% of the mapped reads could be aligned uniquely to a specific subgenome, and around 17% could be mapped to two or all three subgenomes (Supplementary Fig. S2 and Dataset S1). The TGACv1 genome assembly contains 114,428 annotated genes. From our RNA-seq data, we obtained 8,159 assembled transcripts with intact open reading frames corresponding to 3,181 unannotated genes. These putative genes had similar length distribution as that of annotated wheat genes and thus may be real functional genes that have not been annotated (Supplementary Fig. S3, Supplementary Dataset S2 and S3). Therefore, we incorporated these unannotated genes to the total gene set for downstream expression analyses. Wheat genes are distributed equally among the A, B and D subgenomes (A: 35,769 genes, 32.6%; B: 38,535 genes, 35.1%; D: 35,421 genes, 32.3%) (Fig. 2A). After quantifying gene expression levels, 42,173 genes were identified as expressed genes during wheat early spike development. These genes were distributed equally on the three subgenomes (A: 13,920 genes, 33%; B: 14,001 genes, 33.2%; D: 14,252 genes, 33.8%) (Fig. 2A, Supplementary Fig. S4), consistent with the distribution of total annotated genes.

We normalized gene expression levels as fragments per kilobase of transcript per million mapped reads (FPKM)^28^ and found that gene expression levels were generally comparable at the different developmental stages and among subgenomes. However, there was a slightly higher proportion of lowly expressed genes (FPKM < 1) on the B subgenome compared to the A and D subgenomes, suggesting that gene expression was repressed on the B subgenome (Fig. 2B). In each subgenome, the gene expression levels at any two given developmental stages were highly correlated, with the average correlation coefficient (*r*) above 0.98 (Fig. 2C). For instance, a high correlation was observed even between the expression profiles of the vegetative stage and the floret differentiation stage, despite their distinct anatomical and morphological characteristics.

### Dynamic expression of differentially expressed genes in wheat early spike development

**Figure 3:**
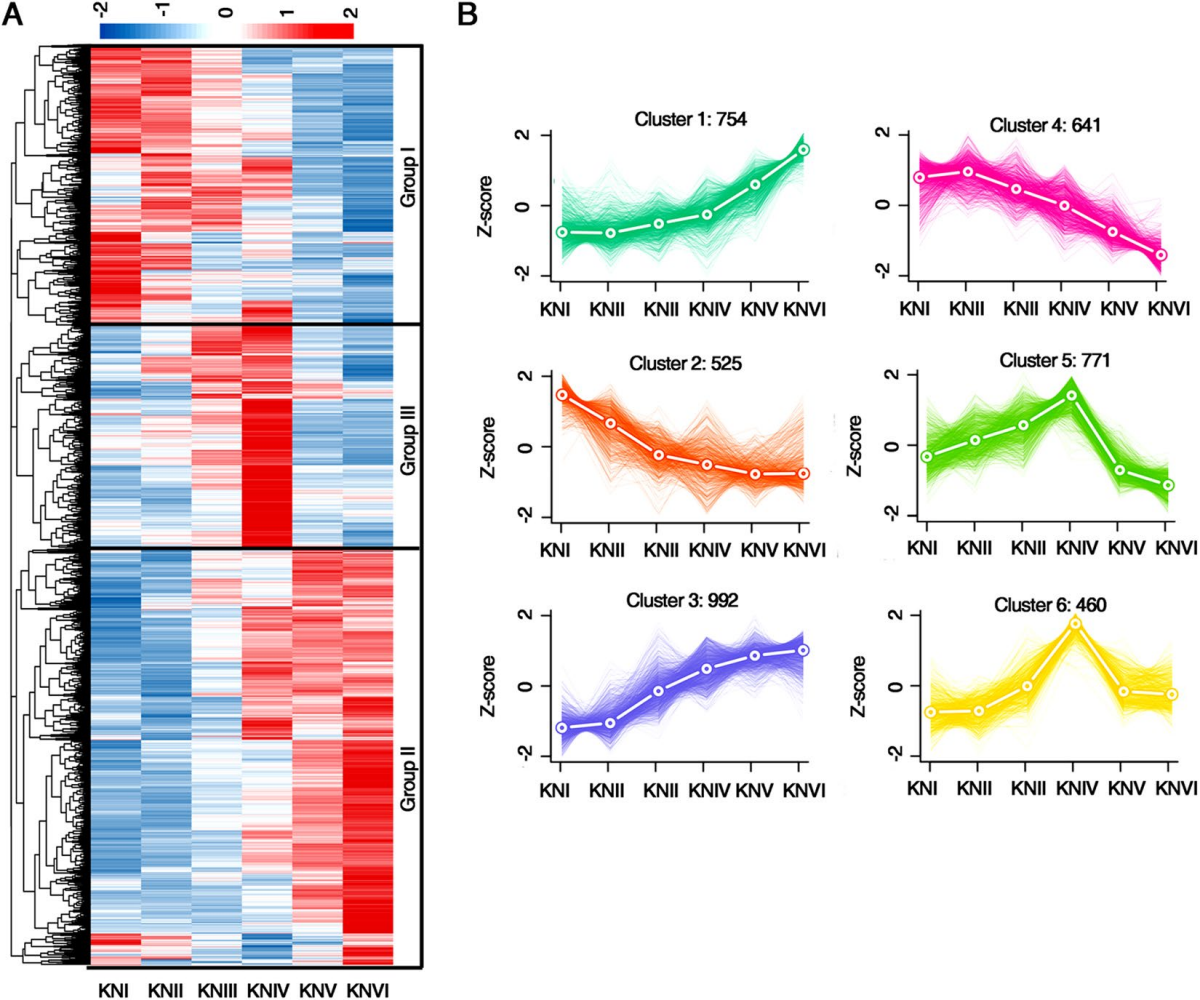
Differentially expressed genes (DEGs) in early wheat spike development. (**A**) Heatmap showing the expression levels of DEGs at six development stages. (**B**) Expression trends of genes in six clusters during the six development stages. Thin lines represent the expression levels of individual genes. Thick dotted lines represent the average expression level of genes in the cluster. The number of genes in each cluster is indicated.

To investigate the expression patterns of genes in early spike development, we identified 4,143 genes as differentially expressed genes (DEGs) in the six tested developmental stages (Supplementary Fig. S5A; Dataset S4) using edgeR (FDR < 0.05 and fold change >2 in normalized expression values)^29^. Among the identified DEGs, 1,272, 1,313 and 1,380 genes (3.0%, 3.1% and 3.3% of expressed genes) came from the A, B and D genomes, respectively, with no indication of subgenome preference (Supplementary Fig. S5B). Hierarchical clustering of the DEGs revealed three groups of genes based on their expression trends from early to late developmental stages (Fig. 3A). While Group I genes had gradually decreased expression, Group II genes exhibited increased expression from early to late stages. These dynamic gene expression patterns are similar to those observed in rice panicle development^30^. In contrast, genes in Group III were predominantly expressed in the middle two stages (KNIII and KNIV), indicating that these genes may regulate the maintenance or phase transition of these stages. Additionally, the heatmaps show that many Group I and Group II genes exhibited a change in the direction of expression between KNIII and KNIV (Fig. 3A). This expression pattern is consistent with the view that the single and double ridge stages are the key stages for the transition from the vegetative stage to the reproductive stage in wheat early spike development^31^.

To explore the possible functions of the DEGs in spike development, k-means clustering analysis was used to further divide the DEGs into six clusters with clear and distinct expression profiles (Supplementary Fig. S5C and Dataset S4). Cluster 1, cluster 2 and clusters 5 and 6 contained genes dominantly expressed in stages KNVI, KNI and KNIV, respectively. Genes in cluster 4 were highly expressed during the first three stages, and cluster 3 contained genes with gradually increased expression during the last three stages (Fig. 3B; Supplementary Fig. S5C). GO analysis was conducted to survey the enrichment of biological process terms in early spike development using topGO for each gene cluster^32^. Overall, genes associated with “intracellular metabolism”; “gene expression regulation and transcription”; and epigenetic mechanisms, such as “histone modification”, “DNA methylation” and “gene silencing by RNA”, were highly enriched (Supplementary Fig. S5D). This analysis indicates that coordinated multilayer biological processes control wheat early spike development, with epigenetic regulation possibly playing a considerable role. Notably, there was significant enrichment for the GO term “regulation of transcription, DNA template” in clusters 3, 4 and 5, indicating a high representation of transcription factor (TF) genes among the DEGs in early spike development (Supplementary Fig. S5D). Interestingly, the GO terms such as “L-phenylalanine catabolic process”, “cellular glucose homeostasis”, “proline catabolic process”, “response to nutrient”, “membrane fusion” as well as “ion transport process” were exclusively enriched in clusters 5 and 6 (Supplementary Fig. S5D). Genes encoding phenylalanine ammonia-lyase (PAL) respond to cold stress^33^ or drought and heat shock^34^. PAL is also a key enzyme in the synthesis of anthocyanin, which plays an important role in chilling and freezing tolerance^33^. Proline and soluble sugars including glucose also have significant roles in freezing tolerance^35,36]^. Additionally, proline has long been considered to participate in the response to osmosis stress and salt stress^37,38^. Overall, numerous genes associated with stress responses and the transport of water, nutrients and ions were highly expressed during the double ridge stage.

Recently, a transcriptome analysis of wheat spike from double ridge stage to tetrads stage was performed^39^. To obtain more information about gene expression change during wheat spike development, we made a combined analysis using our data and theirs. Double ridge stage (KNIV in our study, and DR in theirs) and floret differentiation stage (KNVI in our study, and FM in theirs) were overlapped in the two studies. We calculated DEGs between the two stages (KNIV vs. KNVI and DR vs. FM), respectively. 1464 and 1102 DEGs existed in our and their research, respectively, and 247 overlapped DEGs were found, among which, 220 had the same direction of changes (Supplementary Fig. S6A). The lower proportion of overlapped DEGs might be due to the significant difference of ecotype (winter wheat vs spring wheat) and growth condition (growth room vs field condition) in the two research. GO enrichment of these overlapped DEGs showed that many genes involved in flower development were enriched, suggesting that these genes may be critical for meristem transition (Supplementary Fig. S6B).

### The expression pattern of auxin and cytokinin related genes in spike development

**Figure 4:**
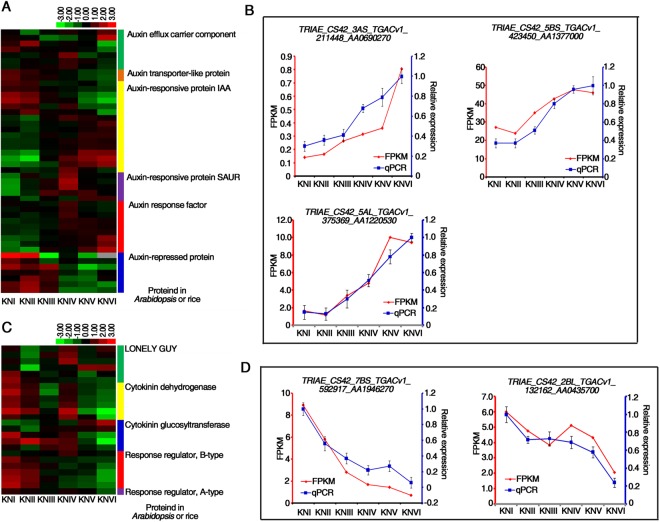
Expression patterns of DEGs in auxin and cytokinin signaling or metabolism pathways in early wheat spike development. (**A**,**C**) Heat map visualizes the expression patterns of DEGs in auxin signalling pathway (**A**) and cytokinin signaling pathway (**C**). Proteins annotated according to homologous genes in *Arabidopsis* or rice are marked by different colors. (**B**,**D**) Side-by-side plot of expression levels of two putative *ARF* genes, *TRIAE_CS42_3AS_TGACv1_211448_AA0690270* and *TRIAE_CS42_5BS_TGACv1_423450_AA1377000*, and one putative *IAA* gene, *TRIAE_CS42_5AL_TGACv1_375369_AA1220530* (**B**), one putative B-type response regulator gene, *TRIAE_CS42_7BS_TGACv1_592917_A A1946270* and one putative A-type response regulator gene, *TRIAE_CS42_2BL_TGA Cv1_132162_AA0435700* (**D**) that were derived from RNA-seq data (red) and verified by RT-qPCR (blue). FPKM: normalized expression levels extracted from RNA-seq data. qPCR: transcript levels measured by real-time RT-PCR. *TaACTIN* served as the internal control. Three biological replicates were performed. Error bars represent SD from three biological repeats. The maximum expression level was set as “1”.

Auxin, one of the primary plant hormones, plays essential roles in axillary meristem (AM) initiation and outgrowth by promoting cell polarity establishment and cell elongation, resulting in a distinct plant architecture, panicle type and grain number per spike^5,40,41^. Components of auxin biosynthesis, transport and signaling have been found to have conserved and distinct roles in the reproductive development of *Arabidopsis*, rice and maize^41,42^, but less progress has been made in wheat in this regard. Among the DEGs, 47 auxin-related genes were identified, including genes encoding AUXIN RESPONSE FACTORs (ARFs), auxin efflux and transport proteins, and auxin responsive proteins such as INDOLE-3-ACETIC ACID (AUX/IAA) proteins and SMALL AUXIN UPREGULATED RNA (SAUR) proteins (Supplementary Dataset S5). Hierarchical clustering of these genes showed that most of genes encoding IAA, SAUR, and ARF proteins were up-regulated from the single ridge stage (KNIII) or double ridge stage (KNIV) onwards (Fig. 4A). Meanwhile, all the genes encoding auxin-repressed proteins had a decreasing trend at the same time (Fig. 4A). We examined the expression changes of two *ARF* genes, *TRIAE_CS42_3AS_TGACv1_211448_AA0690270* and *TRIAE_CS42_5BS_TGACv1_423450_AA1377000*, and one *IAA* gene, *TRIAE_CS42_5AL_TGACv1_375369_AA1220530*, using quantitative PCR and found that the PCR results were highly consistent with the RNA-seq results, indicating that our RNA-seq data were reproducible and reliable (Fig. 4B). Because the expression of this *IAA* gene increases or decreases after auxin induction or depletion^43^, these expression patterns also indicated that auxin signaling was increasing from the single ridge stage. Surprisingly, we failed to detect expression changes in auxin biosynthesis genes, indicating that auxin transport proteins may be critical for auxin distribution during wheat spike development (Fig. 4A).

Cytokinin is a key positive regulator of cell proliferation and organization establishment in the shoot apical meristem (SAM)^44,45^. Among the DEGs identified in the present study, 23 cytokinin related genes, including six homologs of the *LONELY GUY* (*LOG*)^46^ gene in rice, six putative *cytokinin oxidase/dehydrogenase* (*CKX*) genes, five putative cytokinin glucosyltransferase genes, six B-type response regulator (B-type RR) genes and one A-type RR gene, were identified (Fig. 4C and Supplementary Dataset S5). The expression change of *TRIAE_CS42_7BS_TGACv1_592917_AA1946270* gene and *TRIAE_CS42_2BL_TGACv1_132162_AA0435700* were verified by quantitative PCR (Fig. 4D). The former belongs to B-type RR genes, which are positive regulators in the cytokinin signaling pathway^47^ and the later is one of A-type RR genes, which are usually considered as cytokinin marker genes. Besides, this A-type RR gene was included in the homeologous triplet containing *TaRRA12*, whose expression can be induced by cytokinin^48^. The expression change of cytokinin metabolic genes, *CKXs*, *LOGs* and glucosyltransferase genes, had no particular tendencies, while most RR genes were down-regulated from single ridge stage or double ridge stage onwards (Fig. 4C), implying the decreasing of cytokinin signaling from those stages.

### Differentially expressed transcription factor genes in wheat spike development

**Figure 5:**
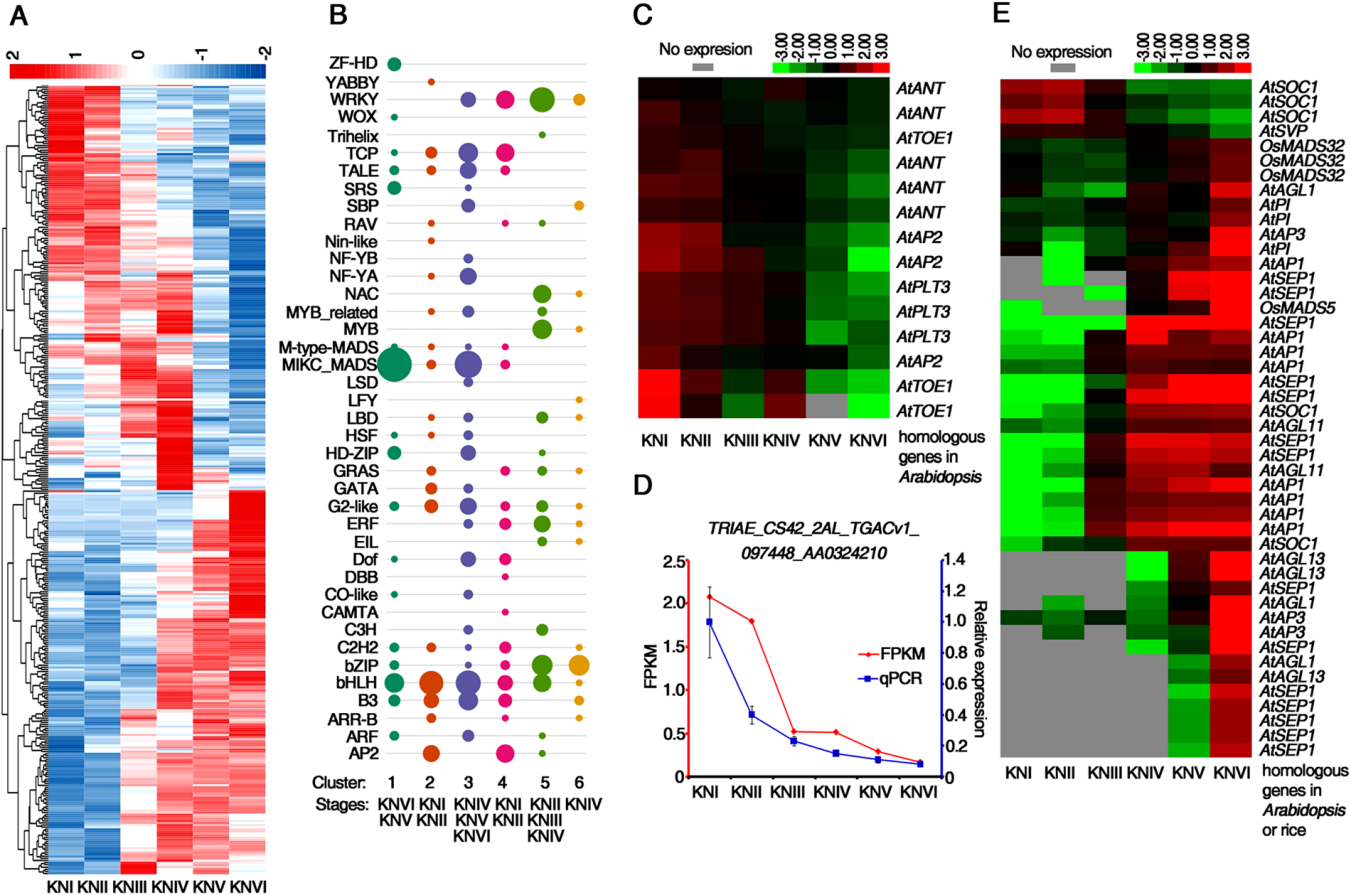
Differentially expressed transcription factor (TF) genes in wheat early spike development. (**A**) Heatmap showing the dynamic expression patterns of the TFs identified as DEGs. (**B**) Number of TF genes that were included in each of the six clusters and enriched stages. Circle size is correlated with number of TF genes. TF family names are listed on the left, and the wheat spike developmental stages at which the genes were strongly expressed are listed on the bottom. (**C**,**E**) Heat map visualizes the expression patterns of AP2 family (**C**) and MIKC-type MADS family (**E**) TFs. (**D**) RT-qPCR validation of the expression patterns at six developmental stages for *TRIAE_CS42_2AL_TGACv1_097448_AA0324210*, a homologous gene to *Arabidopsis AP2*. FPKM: normalized expression levels extracted from RNA-seq data. qPCR: transcript levels measured by real-time RT-PCR. *TaACTIN* served as the internal control. Three biological replicates were performed. Error bars represent SD from three biological repeats. The maximum expression level was set as “1”.

Transcription factors (TFs) have crucial roles in controlling plant growth, development and phase changes by regulating gene expression^49–51^. The enrichment of the GO term “regulation of transcription, DNA-templated” among the DEGs in the present study indicates that TFs also play important roles in wheat spike development (Supplementary Fig. S5D). We identified 375 TF genes with altered expression in our set of DEGs (375 out of 4,143, 9.1%) with distinct expression patterns (Fig. 5A and Supplementary Dataset S6). We categorized the dynamic expression patterns of the various TF gene families by k-means clustering, which yielded six clusters of TF DEGs, each with a distinct gene expression profile (Supplementary Fig. S7). While cluster 1 and 3 genes were highly expressed in KNV and KNVI with an enrichment of MADS domain TFs and bHLH TFs, genes in clusters 2 and 4 were highly expressed in KNI and KNII then subsequently decreased and were enriched for AP2/ERF TFs. Cluster 5 and 6 genes were specifically expressed at the single ridge and double ridge stages during the transition from the SAM to the IM (KNIII and KNIV), with an enrichment of *WRKY*, *bZIP*, *MYB* and *NAC* TF family genes (Fig. 5B).

#### AP2 and MIKC-type MADS transcription factor families

**Figure 6:**
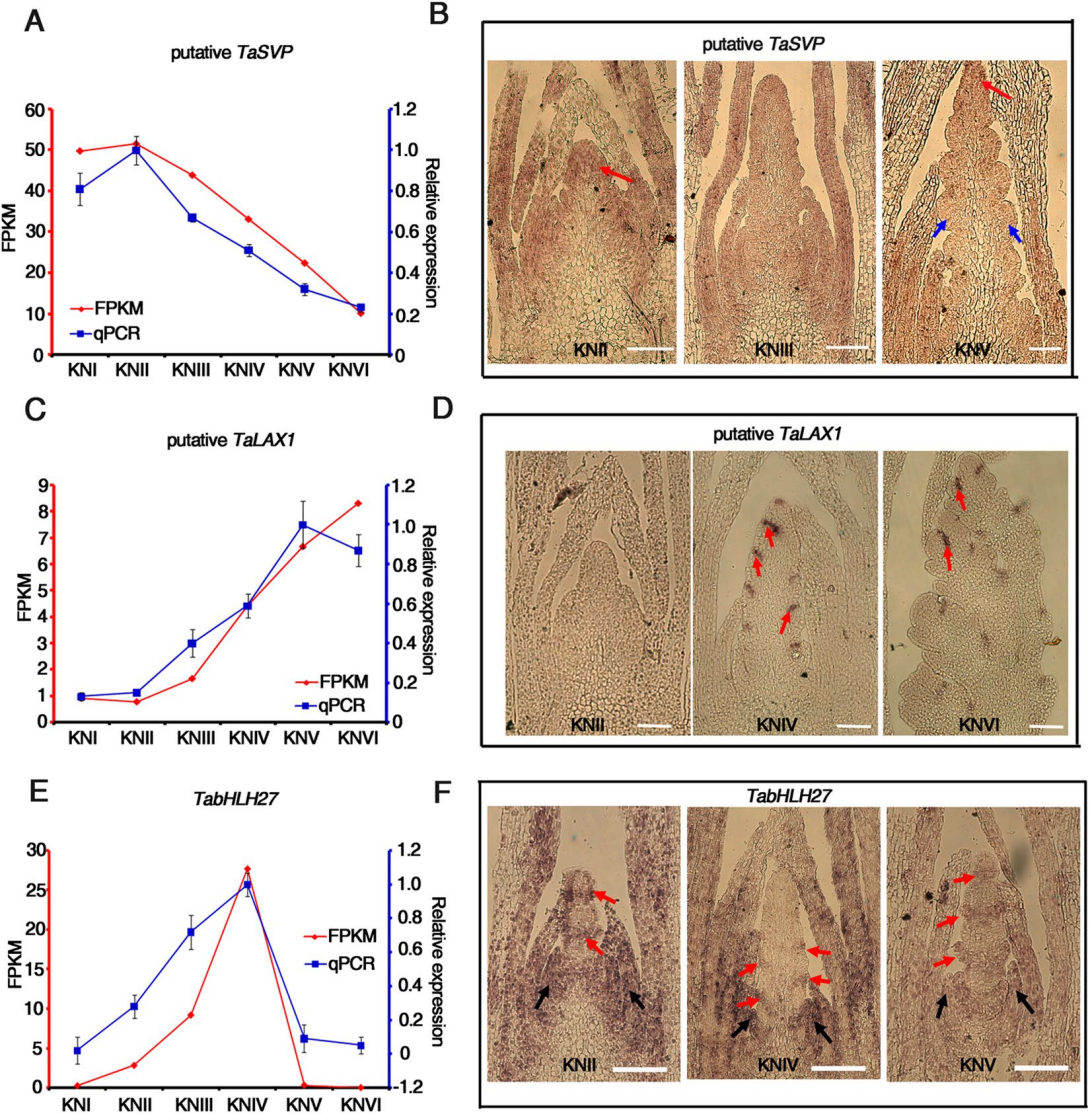
Expression pattern of putative *TaSVP*, *TaLAX1* and *TabHLH27*. (**A**,**C**,**E**) RT-qPCR validation of the expression patterns at six developmental stages for two homologous genes of putative *TaSVP* (A), *TaLAX1* (**C**) and *TabHLH27* (**E**). FPKM: normalized expression levels extracted from RNA-seq data. qPCR: transcript levels measured by real-time RT-PCR. *TaACTIN* served as the internal control. Three biological replicates were performed. Error bars represent SD from three biological repeats. The maximum expression level was set as “1”. (**B**) Expression patterns of putative *TaSVP* by *in situ* hybridization with antisense probe. Signals were detected in inflorescence meristems (red arrows) but not floral meristems (blue arrows). (**D**) Expression patterns of putative *TaLAX1* by *in situ* hybridization with antisense probe. Signals were detected at axillary meristem generating sites and indicated by red arrows. (**E**) Expression patterns of *TabHLH27* by *in situ* hybridization with antisense probe. Signals in leaf primordia were indicated by blue arrows and signals in meristem were indicated by red arrows. Bar: 100 µm in (**D,E,I,J**). Developmental stages were indicated.

The k-means clustering result also showed that the TFs in each cluster came from different families, and TFs belonging to the same family were usually distributed among different clusters (Fig. 5B). This is consistent with previous findings that TFs from one family may participate in different developmental processes and that a given developmental process is regulated by TFs from different families^52^. Compared with other families, TFs from the AP2 and MIKC-type MADS families were more prone to acting similar tendency of expression change (Fig. 5B). Nearly all of the AP2 family TFs had a decreasing expression trend during spike development (Fig. 5C, Supplementary Dataset S7). The expression pattern of *TRIAE_CS42_2AL_TGACv1_097448_AA0324210*, a homolog of the *Arabidopsis AP2* gene, was verified by quantitative PCR (Fig. 5D). Most of the genes homologous to these AP2 TFs in *Arabidopsis* have functions related to “meristem maintenance” or “maintenance of shoot apical meristem identity”. Thus, the higher expression of the AP2 TF genes may contribute to meristem activity maintenance during the first three stages. Most of the MIKC-type MADS TFs were grouped into clusters 1 and 3 (Fig. 6A). Among them, all of the genes homologous to *Arabidopsis AP1* belonged to cluster 3, with increasing expression from KNIII onwards. Most of the genes homologous to *Arabidopsis* floral organ identity genes, such as *AP3* or *SEPALLATA 1* (*SEP1*), had induced expression from KNV or KNVI (Fig. 6D, Supplementary Dataset S7). There were five MIKC-type MADS TFs homologous to *Arabidopsis SUPPRESSOR OF OVEREXPRESSION OF CONSTANS1* (*SOC1*) with opposite expression pattern in DEGs (Fig. 5E, Supplementary Dataset S7). Quantitative PCR was performed to verify the distinct expression patterns of two of the *SOC1* homologous genes. (Supplementary Fig. S8).

#### Spatio-temporal expression pattern of *TaSVP*, *TaLAX1* and *TabHLH27*

To investigate the possible functions of TFs in early spike development, we used *in situ* hybridization to examine the expression pattern of three TFs with distinct expression change, whose homolog had clear function in *Arabidopsis* or rice^53–55^. *TRIAE_CS42_6AL_TGACv1_473223_AA1529480* was homologous to *SHORT VEGETATIVE PHASE* (*SVP*), a gene with the function in repressing flowering in *Arabidopsis*^53^. The transcript levels of the putative *TaSVP* gene continuously decreased according to RNA-Seq data and quantitative PCR (Fig. 6A), and *in situ* hybridization revealed that it was expressed throughout the SAM at the elongation stage (KNII) and concentrated at the tip of the IMs but not in the FMs at KNV (Fig. 6B and Supplementary Fig. 9A–C). These expression patterns are consistent with a role in repressing the floral transition^53^. *TRIAE_CS42_U_TGACv1_641629_AA2099820*, homologous to rice *LAX PANICLE 1* (*LAX1*), with an increasing trend, was verified by quantitative PCR and *in situ* hybridization (Fig. 6C,D and Supplementary Fig. S9D-F). During the KNIV and KNVI stages, *TaLAX1* was specifically expressed in the region where the spikelet and floret primordia were initiated, indicating that it may have the same function in axillary meristem initiation as in rice^54^. *TRIAE_CS42_2BL_TGACv1_129640_AA0391310* (*TabHLH27*), with the highest expression level during double ridge stage was homologous to *At5G57150*, which is involved in dehydration stress in *Arabidopsis*^56^. Its expression pattern was verified by quantitative PCR and *in situ* hybridization (Fig. 6E,F and Supplementary Fig. S9G-I). High but even expression signals were detected in the leaf primordia and very young leaves (Fig. 6F). Unexpectedly, distinct tissue-specific expression signals were also observed at spike meristems. At the elongation stage (KNII), *TabHLH27* was expressed as rings in the meristem although there was no obvious difference between tissues. At the double ridge stage (KNIV), it was clearly expressed at the point of spikelet meristem differentiation (Fig. 6F), indicating potential involvement in the phase transition of meristems and the regulation of meristem differentiation. To see whether the transcriptional expression of *TabHLH27* could respond to the environmental change as its homologs in *Arabidopsis* or rice, we examined its expression level in young spike under normal or adverse growing condition. The result demonstrated that the expression level of *TabHLH27* in young spikes at KNIV stage was dramatically repressed by drought or salt treatments (Supplementary Fig. S10A-B).

### The expression change of *VRN*, *Ppd-1* and *Eps-3*

Several genes regulating spike development in einkorn wheat or bread wheat have been cloned, such as *VERNALIZATION1* (*VRN1*), *VRN2*, *VRN3*, *Photoperiod-1* (*Ppd-1*) and *Eps-3*. Therefore, we analyzed our transcriptome dataset to examine the expression patterns of these genes during early spike development.

**Figure 7:**
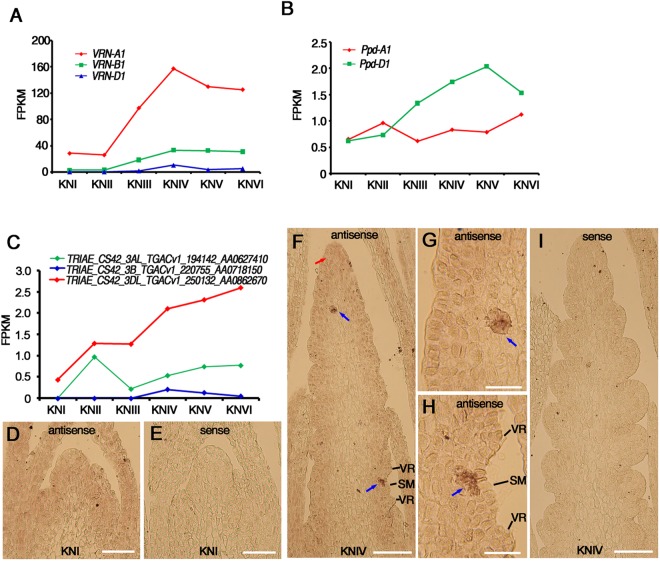
Expression patterns of *VRN1*, *Ppd-1* and *Eps-3*. (**A**) The expression change of three *VRN1* genes during wheat early spike development according to the RNA-Seq data. (**B**) The expression change of two *Ppd-1* genes during wheat early spike development according to the RNA-Seq data. (**C**) The expression change of three *Eps-3* homolog genes during wheat early spike development according to the RNA-seq data. (**D**–**I**) Expression patterns of *Eps-3* at the indicated stages using *in situ* hybridization with antisense probe (**D**,**F**–**H**) and sense probe as control (**E**,**I**). Signals are indicated by a red arrow in the apical region of young spike and blue arrows in the region just beneath the outgrowing spikelet meristems. (**G**,**H**) are magnified figures of the region indicated by the blue arrows in F. SM: spikelet meristem, VR: vegetative ridge. Bars: 100 µm in (**D**–**F**,**I**), 30 µm in (**G**,**H**).

According to a recent study, *VRN-A1*, *VRN-B1* and *VRN-D1* corresponded to *Traes_5AL_13E2DEC48*, *Traes_5BL_5D2D22E67* and *Traes_5DL_9CC4EC839* in bread wheat, respectively^57^. The transcriptome analysis result showed that all the three *VRN1* genes were highly expressed from KNIV to KNVI (Fig. 7A). with the expression level of *VRN-A1* being significantly higher than that of *VRN-B1* or *VRN-D1* (Fig. 7A). For other two *VRN* genes, *VRN2* and *VRN3*, no appreciable expression was detected throughout wheat early spike development.

The *Ppd-D1* gene, *TRIAE_CS42_2DS_TGACv1_179266_AA0605730*, which modulates spike architecture in bread wheat^58^, showed higher expression level from KNIII to KNVI. While *Ppd-A1* gene, *TRIAE_CS42_U_TGACv1_644248_AA2138300*, was expressed constantly (Fig. 7B). Different from *Ppd-D1* and *Ppd-A1*, *Ppd-B1* (*TRIAE_CS42_2BS_TGACv1_147969_AA0489460*) did not show appreciable expression throughout early spike development.

Three homologous genes of *Eps-3* that modulate narrow-sense earliness and spikelet number in einkorn wheat^59^ were found in the dataset, with the D copy having significant dominance in the homologous triplets (Fig. 7C). *In situ* hybridization demonstrated that *Eps-3* had a weak signal in the whole SAM and young leaves during KNI (Fig. 7D,E), a stronger signal in the apical region of IM and a distinct tissue-specific signal beneath the outgrowing SM during KNIV (Fig. 7F–I). This expression pattern suggests that *Eps-3* functions in the transition process from IM to SM.

### DEGs between the SAM and IM in wheat and rice during the floral transition

**Figure 8:**
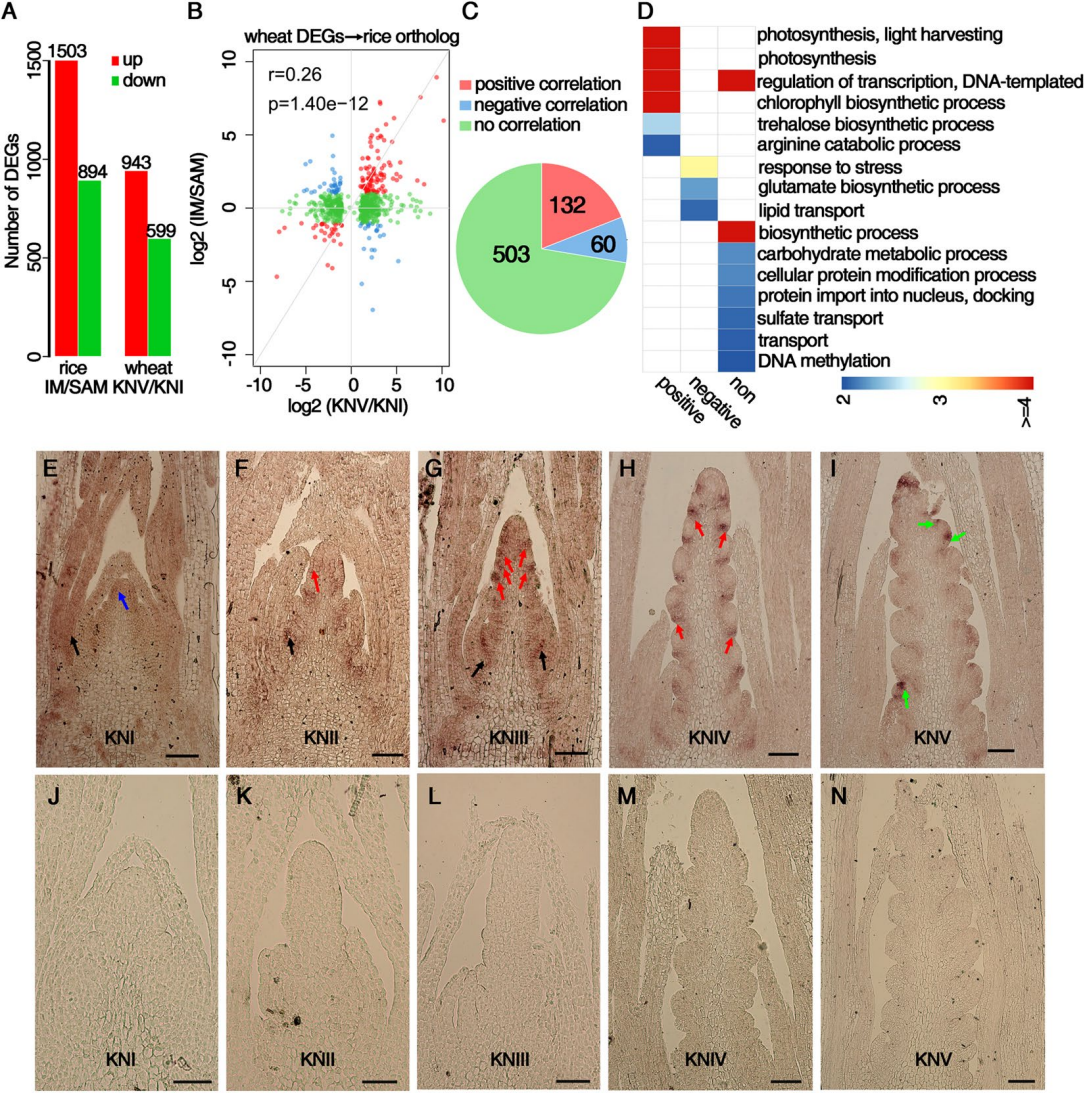
Comparison of differentially expressed genes (DEGs) between the inflorescence meristem (IM) and shoot apical meristem (SAM) during the floral transition in wheat and rice. (**A**) Number of DEGs between the IM and SAM in rice and number of DEGs between the glume primordium differentiation stage (KNV) and floret differentiation stage (KNI) in wheat spike. (**B**) Scatter plot of expression level fold change of rice and wheat homologous genes. Red, blue and green dots indicate positive, negative and no correlation, respectively. r, Pearson correlation coefficient; p, p-value. (**C**) Number of rice and wheat homologous genes with positive (red), negative (blue) or no (green) correlation in expression. (**D**) GO terms that are enriched in rice homologous genes with positive, negative or no correlation. Each square is colored according to the –log_10_(p) value, where p is the p-value for the significance of GO term enrichment. (**E**–**N**) Expression patterns of *WFL*, the *LEAFY* homolog in wheat, at the indicated stages using *in situ* hybridization with antisense probe (**E**–**I**) and sense probe as control (**J**–**N**). Signals are indicated by black arrows in leaf primordia, red arrows in bracteal primordial, blue arrows at the whole SAM and green arrows at the floral meristem. Scale bar: 100 µm.

Several previous studies have used transcriptome analysis to investigate rice panicle development^30,60^. To compare the function of genes involved in the floral transition in wheat and rice, we downloaded rice SAM and IM RNA-seq data published in a previous study in which the rice SAM and IM were collected separately and DEGs were identified^60^. To compare the published dataset with our own, we analyzed the rice RNA-seq data using our workflow described above and our more stringent criteria for DEGs (FDR < 0.05 and fold change >2). We found 1,503 and 894 up- and down-regulated genes, respectively, in the rice IM compared with the SAM, comparing to the 1,616 and 1,010 genes in Liu’s research^60^ (Fig. 8A and Supplementary Dataset S8). Anatomically and morphologically, the rice SAM and IM correspond to the samples we collected in KNI and KNV, respectively. We found 943 and 599 up- and down-regulated genes, respectively, in KNV compared to KNI, which we subsequently compared with the rice DEGs (Fig. 8A). Firstly, we aligned all of the DEGs from wheat with the rice database and obtained 695 best homologs in rice for these wheat DEGs (for this purpose, wheat triplets were considered as a single gene). Expression level fold change analysis showed a high correlation for the homologs from these two species (*r* = 0.26, *p* = 1.40e-12) and identified 132, 60 and 503 genes with positive correlation, negative correlation and no correlation, respectively (Fig. 8B,C; Supplementary Dataset S9). GO analysis revealed that “photosynthesis process”, “transcription regulation”, “chlorophyll biosynthetic process” and “trehalose biosynthetic process” terms were significantly enriched among the positively correlated genes, indicating that these biological processes are conserved in the spike development of these two species. Among the negatively correlated genes, “response to stress” was highly enriched (Fig. 8D). Secondly, we identified 965 wheat best homologs of rice DEGs and performed the same analysis described above. A high correlation for the expression patterns (r = 0.25, p = 8.64e-16) was still detected, and genes related to photosynthesis, transcription regulation and chlorophyll biosynthesis were over-represented among the positively correlated genes (Supplementary Fig. S11).

To dissect the potentially different roles of genes in wheat and rice flowering, we used *in situ* hybridization to investigate the expression pattern of *TRIAE_CS42_2DL_TGACv1_159321_AA0536540*, the wheat homolog of *Arabidopsis LEAFY* and rice *ABERRANT PANICLE ORGANIZATION2*/*RFL* (*OsAPO2*/*RFL*). The wheat homolog has been reported as *WHEAT FLORICAULA*/*LFY* (*WFL*)^61,62^. In *Arabidopsis*, *LFY* is expressed in floral buds and encodes a plant-specific transcriptional regulator that determinates FM identity and promotes meristem determinacy^63,64^. At KNI (vegetative stage), a weak *WFL* signal was detected throughout the SAM and leaf primordial (Fig. 8E,J). From KNII to KNIV, the expression of *WFL* was specifically at the vegetative region or the region where the vegetative ridge would be initiated, but absent at the spikelet meristem (Fig. 8F–H,K–M), consistent with the expression pattern of *OsAPO2*/*RFL* in rice, except that *WFL* was not expressed at the spike apex where a terminal spikelet would be generated^61,65^. At KNV, *WFL* signal was observed at the floral meristem region in lateral spikelets, a pattern similar to *RFL* expression in rice (Fig. 8I,N)^65^.

## Discussion

The spikelet number, which can dramatically influence grain number in wheat, is determined before the formation of terminal spikelet in floret differentiation stage. A systematic study of gene expression change is helpful to uncover the molecular mechanism of wheat early spike development and provide valuable resources for the improvement of grain number.

### The phase transition in wheat early spike development is controlled by a small subset of important genes

From vegetative stage to floret differentiation stage, the young spike undergoes the transition from SAM to IM, and the initiation of spikelet meristem and floral meristem, sequentially. This process may need several months for typical winter wheat growing in the field. Despite the distinct anatomical and morphological characteristics among different stage of early spikes, the correlation coefficient was extremely high, and only decades or hundreds DEGs were found between any two given stages when it grown in greenhouse after vernalization according to our study (Fig. 2C and Supplementary Fig. S5A). This characteristic was also found by Feng, in which more than10,000 DEGs were identified between the meristematic tissues and the young florets, but only 753 DEGs were found between double ridge stage (KNIV) and floret meristems (KNVI)^39^. As expected, correlation coefficients were higher for closer developmental stage pairs^66^. These findings suggest that the expression profiles of most genes remain fairly stable during wheat early spike development, except for a small set of genes involved in the initiation and transition of different stages.

### The expression dynamics of spike development regulatory genes (*VRN*, *Ppd-1* and *Eps-3*)

For wheat development, the timing of heading is usually controlled by vernalization requirement, photoperiod sensitivity and narrow-sense earliness (earliness per se)^67^. Besides modulating flowering time, most of genes related to these processes have pleiotropic effects, including regulating spike development^67,68^. Significant expression of several such genes were observed in our transcriptome dataset. For instance, as described earlier, the *VRN1* genes, which function in vernalization requirement, were highly expressed from KNIV to KNVI (Fig. 7A), consistent with a previous report that *VRN1* was expressed more highly in young spike compared with shoot apical apex after full vernalization^69^. Moreover, *VRN-A1* had higher transcription level than *VRN-B1* and *VRN-D1* (Fig. 7A), which agrees with the finding in a previous study and the stronger effect of *VRN-A1* in reducing the vernalization requirement among the triplet^70,71^. On the other hand, the other two vernalization related genes, *VRN2* and *VRN3*, did not show appreciable expression during early spike development. The silencing of *VRN2* was consistent with the observation that the expression of *VRN2* could be significantly repressed by vernalization^72^. Meanwhile, *VRN3*, a homologous gene of *Arabidopsis FT* that was expressed in leaves and its protein was transported to the shoot apical meristem, was also not detected in young spike in a recent study^57^. The *Ppd-D1* gene was expressed higher during the stage when spikelets were differentiated (Fig. 7B), consistent with its function in modulating paired spikelet development in bread wheat^58^. The expression of *Eps-3* was initiated when apical meristem changed from double ridge stage to the next phase of spikelet differentiation (Fig. 7D–I). This agreed with its function in regulating narrow-sense earliness (earliness per se)^59^. Given that the materials we used for RNA-seq analysis were grown under long photoperiod after full vernalization, this expression pattern indicated that *Eps-3* can modulate the timing of heading when the vernalization requirement and photoperiod were all optimal just as in a previous report^59^. The specific expression of *Eps-3* at the apical region and the region just beneath the outgrowing SM (Fig. 7D–I) indicated its function in the transition process from IM to SM, and this also agreed with a previous report that *Eps* genes can modulate flowering time by regulating the initiation of floral primordial^68^.

### The conservation and divergence of homologous genes between wheat and other species

The architecture of inflorescence of wheat is significantly different from other species, such as *Arabidopsis* or rice. The expression pattern and function of homologous genes may be conserved or diverged among different species. Our gene expression profile demonstrated that many key regulatory genes may be conserved between wheat and *Arabidopsis* or rice. In *Arabidopsis*, *SVP*, a flowering repressing gene, and *SOC1*, a flowering promoting gene, had a decreasing and increasing expression pattern from vegetative to reproductive stage, respectively^53,73^. Consistently, the expression level of *TaSVP* and two *SOC1* homologous genes decreased and increased, respectively, in wheat early spike development (Figs 5E, 6A and Supplementary Fig. S6A), implying their similar functions in the regulation of flowering time. Additionally, genes homologous to *AP3* and *SEP1* specifically expressed during glume primordium differentiation stage or floret differentiation stage (Fig. 5E), demonstrating conserved function in the modulation of floral organ differentiation as that in other species^7^. The similar spatial expression pattern of *LAX1* in wheat and rice suggested *TaLAX1* may also functions in the regulation of axillary meristem initiation (Fig. 6D)^54^.

Besides these conserved genes, several genes with diverged expression pattern between wheat and other species were also found. For instance, the three *SOC1* homolog genes with decreasing expression during spike development may have functional divergence in the modulation of flowering time (Fig. 5E and Supplementary Fig. S6B). *AP1*, the floral organ identity gene in *Arabidopsis*, had no function in modulation vernalization requirement^74^. But in wheat, its homologs were important genes involved in vernalization requirement^69^. In rice normal panicles, the inflorescence meristem eventually aborts after several primary branches differentiate, and a vestige of the meristem rather than a terminal spikelet is observed^65,75^. Conversely, the inflorescence of wheat is determinate, with the inflorescence meristem converted into a terminal spikelet. As with *OsAPO2*/*RFL* prior to the initiation of spikelets in rice panicle branches, its homolog *WFL* was not expressed at the regions where the spikelet meristem was initiated in wheat, indicating that the two homologs have similar functions in repressing spikelet meristem differentiation (Fig. 8H). However, *OsAPO2*/*RFL* was shown to be expressed at the panicle apex^61^, whereas *WFL* was not (Fig. 8H). This difference may be associated with the divergence in inflorescence determinacy between rice and wheat.

### Possible function of auxin and cytokinin in wheat early spike development

Auxin and cytokinin are key regulators during meristem development. According to our gene expression profiles, auxin and cytokinin signaling increased or decreased throughout the wheat early spike development, respectively (Fig. 4A,C). Increased auxin signaling may promote the production of numerous AMs at the spikelet and floret differentiation stage, consistent with the function of auxin in AM initiation and outgrowth. The higher cytokinin signaling level before double ridge stage is consistent with its function in maintaining meristem activity to suppress the precocious conversion of IM to SM, and to ensure the formation of enough number of spikelets^46,76^. The interaction of cytokinin and auxin is essential for the maintenance of SAM. While cytokinin promotes the proliferation of stemness cells in the SAM, auxin induces cellular differentiation and organ outgrowth^77^. In the present analysis of wheat spike development, we also detected coordinated changes in auxin and cytokinin activity. In contrast to the higher cytokinin signaling level before the double ridge stage, which may help repress cell differentiation and ensure the meristem activity of the SAM, auxin activity was dramatically up-regulated from the double ridge stage onwards, most likely contributing to the generation of new axillary meristems (Fig. 4A,C).

### Possible function of transcription factor in wheat early spike development

Transcription factors are also key regulators of wheat early spike development. It may be involved in meristem maintainence (such as AP2 family TFs), flowering time modulation (such as *TaSVP*, *AP1* and *SOC1* homologs), regulation of meristem initiation or transition (such as *WFL*, *TaLAX1* and *Eps-3*), or regulation of floral organ development (such *AP3* and *SEP1* homologs). Besides, TFs like *TabHLH27*, which have specific expression pattern in young spike and respond to stress (Fig. 6F and Supplementary Fig. S10), may be involved in the coordinated process between stress response and spike development and is therefore a good candidate for crop improvement.

### Dual functionality of stress response genes in wheat early spike development

To survive from the abiotic stress, land plants have evolved complicated adaptation systems that include developmental, physiological and biochemical changes regulated by stress-response genes expression^78–81^. A recent study showed that genes involved in stress and defense signaling was the only enriched biological function in DEGs between two near isogenic lines with distinct spike type in barley^82^, demonstrating the potential function of stress related genes in the regulation of spike development. Indeed, numerous genes associated with stress responses and the transport of water, nutrients and ions were found with the highest expression at the double ridge stage (Supplementary Fig. S5). Unexpectedly, some genes, such as *TabHLH27*, had distinct expression patterns in leaf primordia and SAM, respectively, showing ubiquitous expression in the leaf but growing point-specific expression in SAM (Fig. 6E), which indicated the communication and interaction between the young spike and the environment. Since the gene expression profiling was carried out under constant environment in growth room, many stress-related genes expressing in the early spike suggested that spike development might necessitate the expression of stress-related genes independent of environment stressors. Despite all that, the expression level of these stress-related genes might be influenced by environment, which would finally affect the spike development just like *LATERAL ORGAN BOUNDARIES DOMAIN12-1* (*LBD12-1*) which could transduce salt signal to SAM development in rice^83^. Previous studies showed that many environmental factors, such as temperature, photoperiod, water and minerals, regulate the rate and duration of spikelet differentiation, apical spikelet formation and consequently, final grain number^84–87^. Since most of the stress response gene were highly expressed during double ridge stage when most of spikelets are differentiated (Supplementary Fig. S5)^88^, we hypothesized that these stress-response genes may coordinate the crosstalk between environmental signals and the developmental process, and then be good candidates for the improvement of grain number per spike in wheat through genetic engineering.

## Materials and Methods

### Plant materials

The winter wheat variety Kenong 9204 (KN9204) was used in this study and reported previously^23,24^. The germinated seeds were treated at 4 °C for 40 days. The seedlings were transplanted into soil and grown in the growth room at 22 °C under long day conditions (16 h light/8 h dark). The stage-specific spikes of wheat were dissected under the stereomicroscope based on the anatomic and morphological features as previously described^8^ during 10:00 AM to 12:00 PM of the day. About 200 spikes (for KNI to KNIII) or 100 spikes (for KNIV to KNVI) were pooled to make each RNA sample. Two biological replicates were collected for the RNA-seq analysis of the spikes at each of the six development stages.

To detect the expression change of *TabHLH27* gene under drought or salt treatment, plants were grown as above except that they were not irrigated from KNI to KNIV (drought stress), or irrigated with 100 mM NaCl until KNIV (salt stress).

### RNA extraction, quantitative PCR and *in situ* hybridization

Total RNA was isolated using the RNeasy plant mini kit (Qiagen) and treated with DNaseI (Roche) to eliminate contaminating DNA as previously reported^89^. For qPCR verification, three biological replicates, independent of RNA-seq samples, were collected and assayed. M-MLV Reverse Transcriptase (Promega) was used for reverse transcription. Quantitative real-time RT-PCR was performed in triplicate on the Biorad CFX-96 Real-time PCR system (Biorad) using the SYBR Green RT-PCR kit (DBI Bioscience). The primers used for qPCR were listed in Supplementary Table S1.

*in situ* hybridization was performed as previously described^89^. The primers corresponding to tested genes were added by T7 and SP6 promoter sequence for antisense and sense probe, respectively. *in vitro* transcription was conducted with either T7 or SP6 RNA polymerase to generate the antisense or sense probe using the purified PCR product as the template. The primers used for probe synthesis were listed in Supplementary Table S1.

### RNA-seq and data analysis

#### RNA-seq

RNA-seq libraries were constructed using TruSeq Stranded mRNA Library Prep Kit (Illumina) to generate Illumina sequencing libraries according to the manufacturer’s instructions. Paired-end sequencing was performed on an Illumina HiSeq2500 sequencer.

### Preprocessing, mapping and filtering of RNA-seq reads

We cleaned the paired-end reads by Trimimomatic^90^. After trimming the adapter sequence, removing low quality bases and filtering short reads, clean reads were mapped to the latest *Triticum aestivum* genome assembly (TGACv1, Ensembl Plants) by TopHat^91^ version 2.1.1 with default parameters. The aligned reads were filtered using a set of rules described previously^27^ and classified into six groups (Supplementary Fig. S2). Only uniquely aligned reads were retained for downstream analyses.

### Transcriptome assembly and discovery of unannotated transcripts

We merged the filtered alignments of the two biological replicates for each sample and performed Cufflinks^92^ version 2.2.1 to assemble the transcripts with TGACv1 gene models as a guide. After assembling transcripts for six samples, we combined them into a master transcript set and compared it to the annotated transcripts using Cuffcompare^92^ version 2.2.1. We selected the unannotated intergenic transcripts whose class code was labeled as “u” in the Cuffcompare output, and predicted the coding regions within those transcripts using TransDecoder^93^ version 3.0.0. We used Blast2GO^94^ to assigned GO terms to the unannotated transcripts. The unannotated transcripts with a complete open reading frame (ORF) were added to the annotated transcript models for further analysis.

### Identification of differentially expressed genes and clustering analysis

Number of reads that were mapped to each gene was calculated with the *htseq-count* script in HTSeq.^95^. Normalized expression value (FPKM, Fragments Per Kilobase of transcript per Million mapped reads) for each gene was calculated by Cuffdiff^92^ version 2.2.1. EdgeR^29^ was used to identify genes that were differentially expressed between each pair of samples. Genes with at least two-fold change in expression and a false discovery rate (FDR) less than 0.05 were considered differentially expressed genes (DEGs). Hierarchical and K-means clustering was applied to the DEGs by pheatmap^96^.

### Expression of orthologous genes in *Oryza sativa*

BLASTP (e-value cutoff was set to 1e-5) was performed to identify orthologous genes between *Triticum aestivum* (TGACv1) and *Oryza sativa* (MSU7.0). The best hit of a query sequence was considered as the orthologous gene. The RNA-seq data of rice inflorescence meristem (IM) and shoot apical meristem (SAM)^60^ were downloaded from the Gene Expression Omnibus (GEO) database (accession number GSE68299) and mapped to the genome of *Oryza sativa* by TopHat. The calculation of read counts and FPKM for each gene and the identification of DEGs were performed in the same way as we did for wheat data.

To assess the correlation of the DEGs between wheat and rice during spike development, we compared the log_2_[fold-change between glume primordium differentiation stage (KNV) and floret differentiation stage (KNI)] of DEGs in wheat spike to the log_2_(fold-change between IM and SAM) of orthologous genes in rice. Inversely, we also compared the log_2_(fold-change between IM and SAM) of DEGs in rice to the log_2_(fold-change between KNV and KNI) of orthologous genes in wheat.

### Transcription factor families

The protein sequences of *Triticum aestivum* transcription factors (TFs) were downloaded from Plant Transcription Factor Database^97^. These TF sequences were based on wheat genome assembly version IWGSC. We used BLASTP to identify the corresponding proteins in the latest TGACv1 assembly.

### Gene Ontology analysis

Gene Ontology (GO) terms of *Triticum aestivum* genes were taken from Ensembl BioMarts^98^. The GO enrichment analysis was performed with topGO^32^. The GO terms with a p-value below 0.05 were considered significantly enriched.

## Electronic supplementary material

Supplementary Figures S1-S11

Dataset 1

Dataset 2

Dataset 3

Dataset 4

Dataset 5

Dataset 6

Dataset 7

Dataset 8

Dataset 9

Dataset 10

## Data Availability

All RNA-seq data have been deposited in the Gene Expression Omnibus (GEO) database under accession numbers GSE83287. The sequences of genes we discussed have been deposited in GeneBank under the accession numbers: TaLAX1 (KY495616), TabHLH27 (KY495618), TaSVP (KY495619).
