# Supplementary material for: A Genome-wide View of Transcriptome Dynamics During Early Spike Development in Bread Wheat
Source: Sci Rep. 2018 Oct 18;8:15338. doi: 10.1038/s41598-018-33718-y (PMC6194122; doi:10.1038/s41598-018-33718-y)
Supplement: Supplementary file 1 — Supplementary Figures S1-S11 [file 41598_2018_33718_MOESM1_ESM.pdf]

A Genome-wide View of Transcriptome Dynamics During Early Spike Development in Bread Wheat

Yongpeng Li, Xing Fu, Meicheng Zhao, Wei Zhang, Bo Li, Diaoguo An, Junming Li, Aimin Zhang, Renyi Liu, Xigang Liu

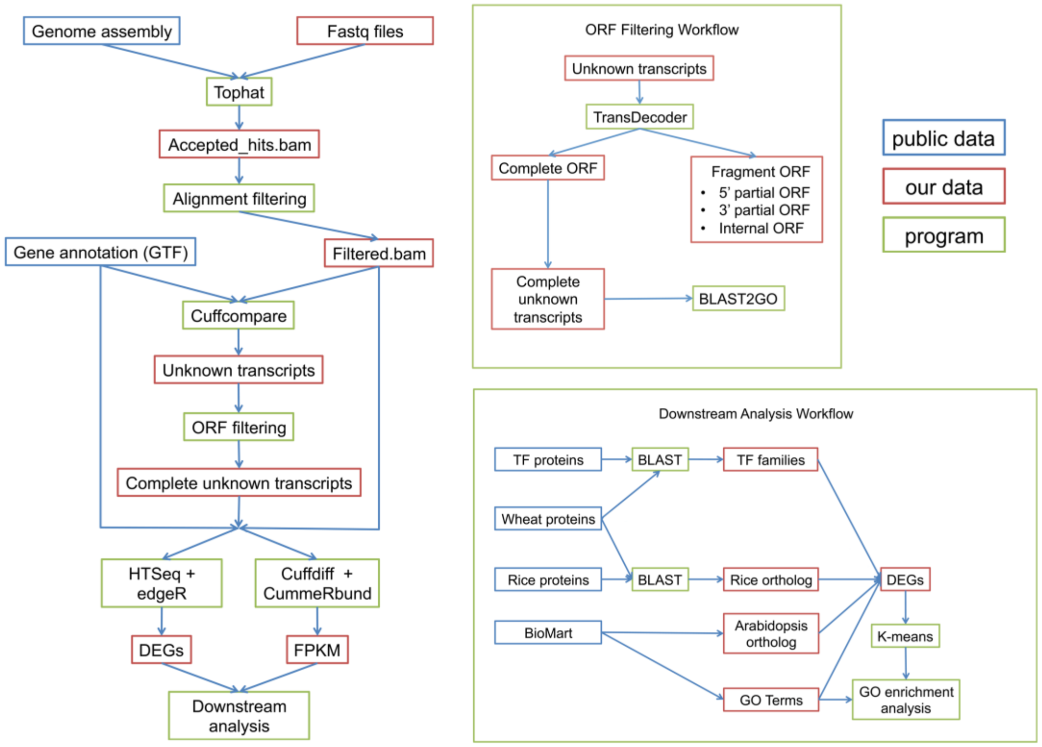

Figure S1. Schematic illustration of the bioinformatics analysis work flow.

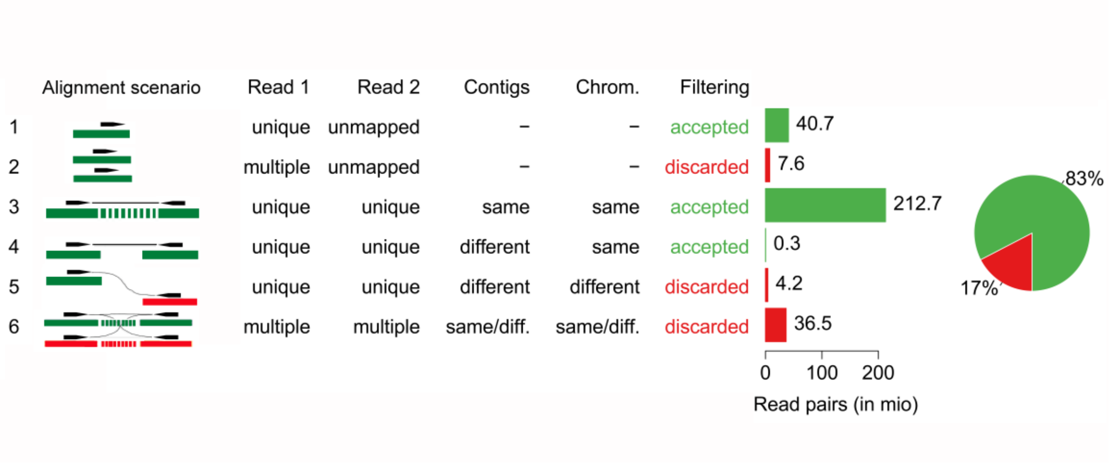

Figure S2. Classification of RNA-seq read pair mappings to six alignment scenarios for stringent read filtering. The aligned RNA-seq reads were categorized into six groups and filtered. The bar chart shows the number of read pairs assigned to the corresponding groups. The pie chart shows the overall proportions of paired-end reads that were accepted (green) and discarded (red). Only uniquely mapped reads or read pairs were retained for downstream analyses.

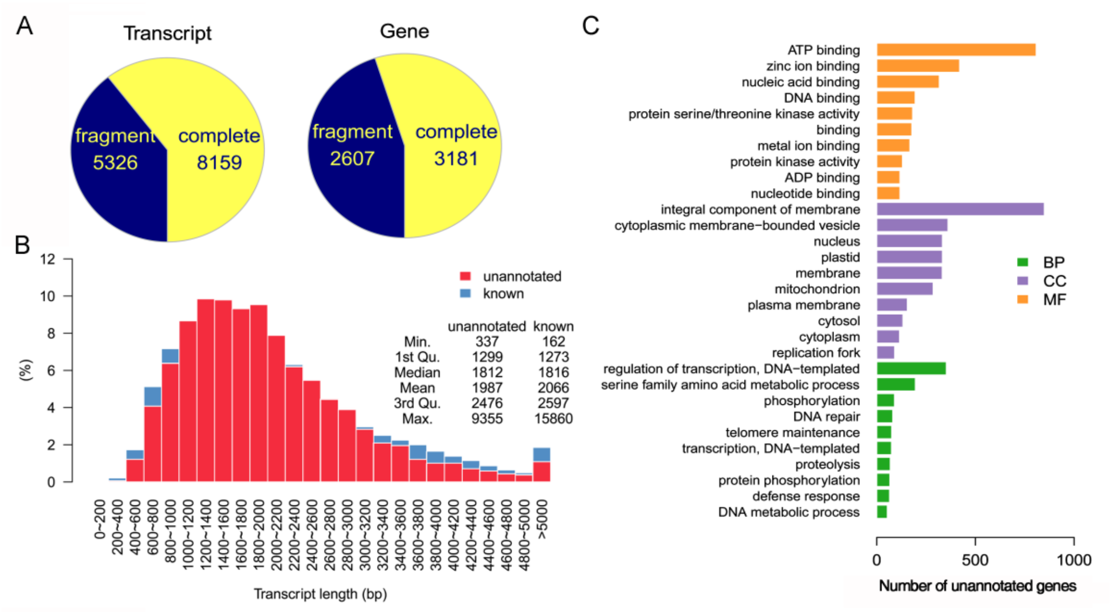

Figure S3. Discovery of unannotated genes using RNA-seq data in six wheat spike developmental stages. (A) Number of newly assembled transcripts and genes whose coding regions were predicted and classified as fragmented or complete by TransDecoder. (B) Length distribution of annotated transcripts in the wheat genome and newly assembled transcripts. As shown in the figure, the two transcript sets have similar length distributions. (C) GO terms assigned to the newly discovered unannotated genes by Blast2GO; the number of genes is indicated on the x-axis. The top ten GO terms in each category are shown in the bar plot. BP: biological process; CC: cellular component; MF: molecular function.

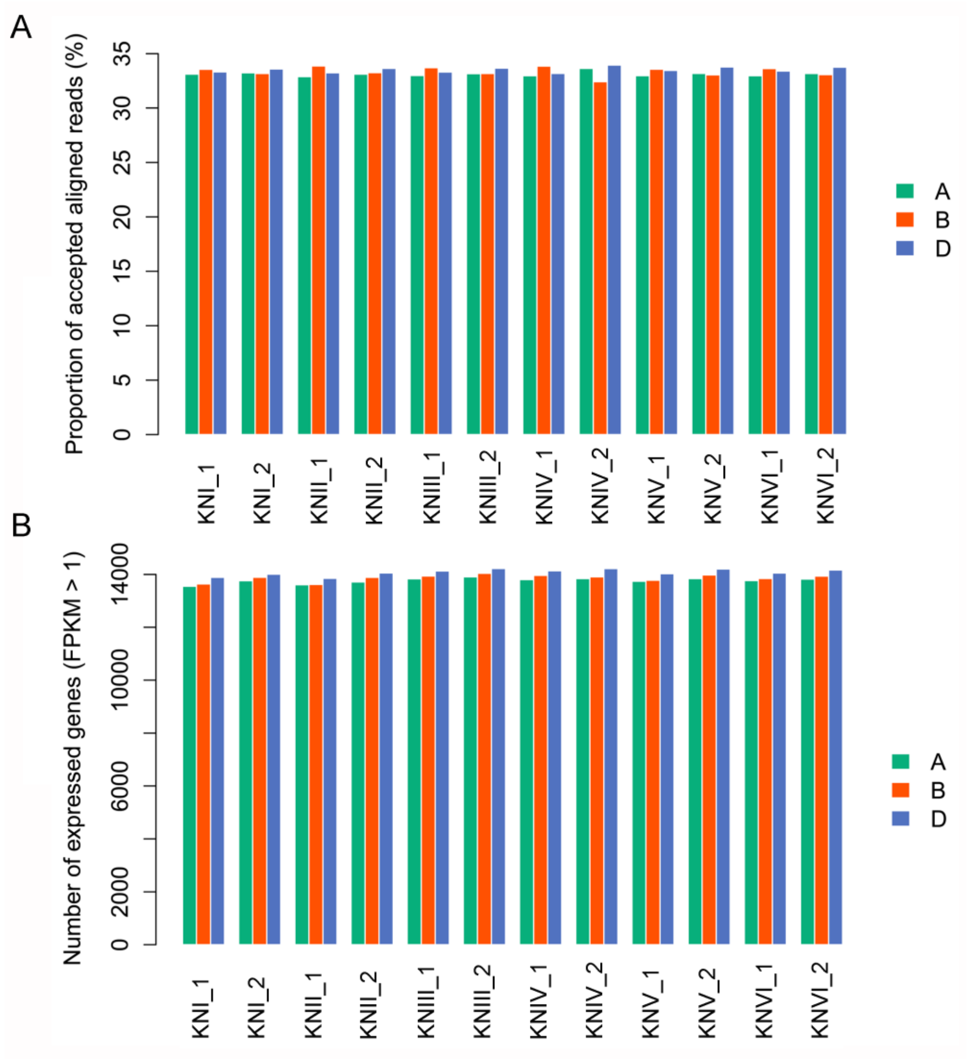

Figure S4. Proportion of reads (A) and number of expressed genes (B) that were attributed to the three subgenomes in each RNA-seq sample.

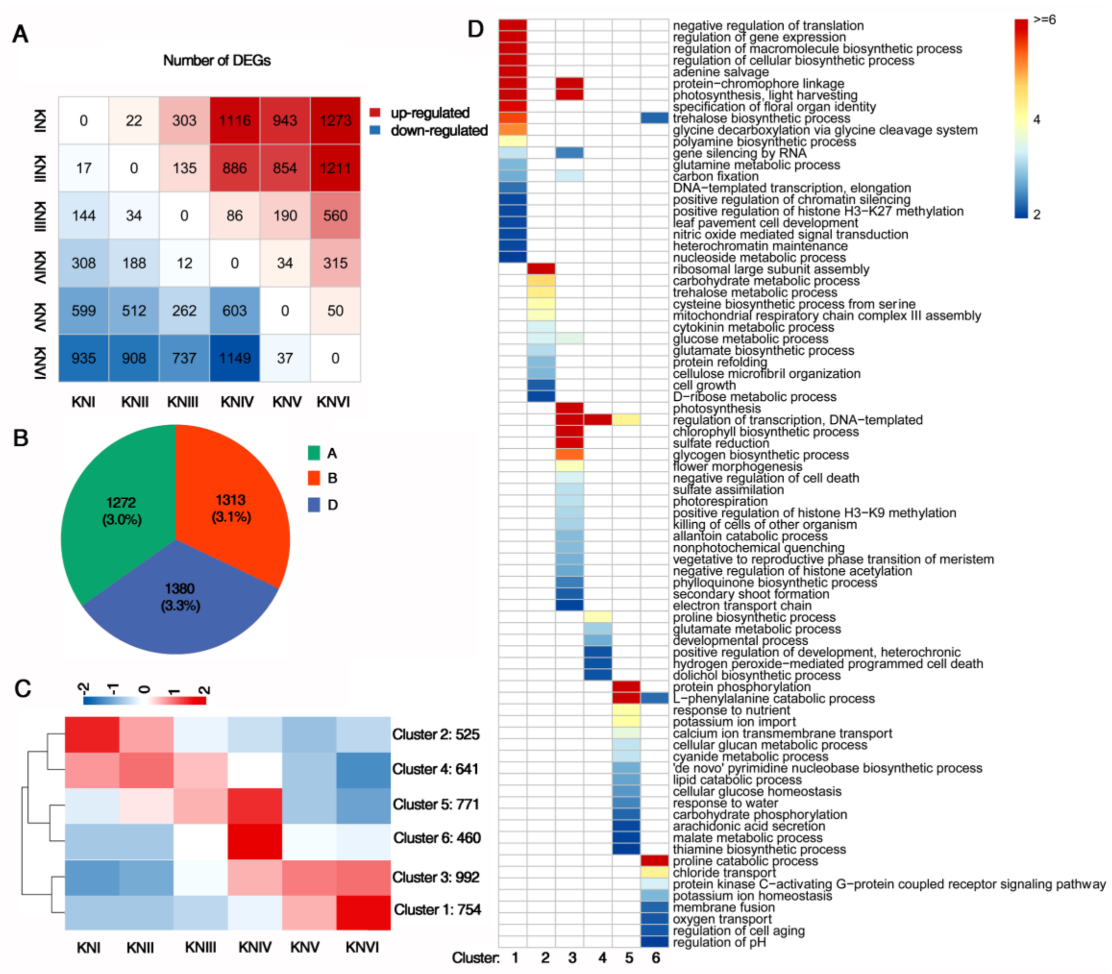

Figure S5. The k-means clusters of differentially expressed genes (DEGs) in early wheat spike development. (A) Number of DEGs between each pair of development stages. (B) The number and proportion of DEGs located on the A, B, and D subgenomes. (C) Heatmap showing the average gene expression trend for six clusters during the different development stages. (D) GO terms that were enriched among genes in the six clusters.

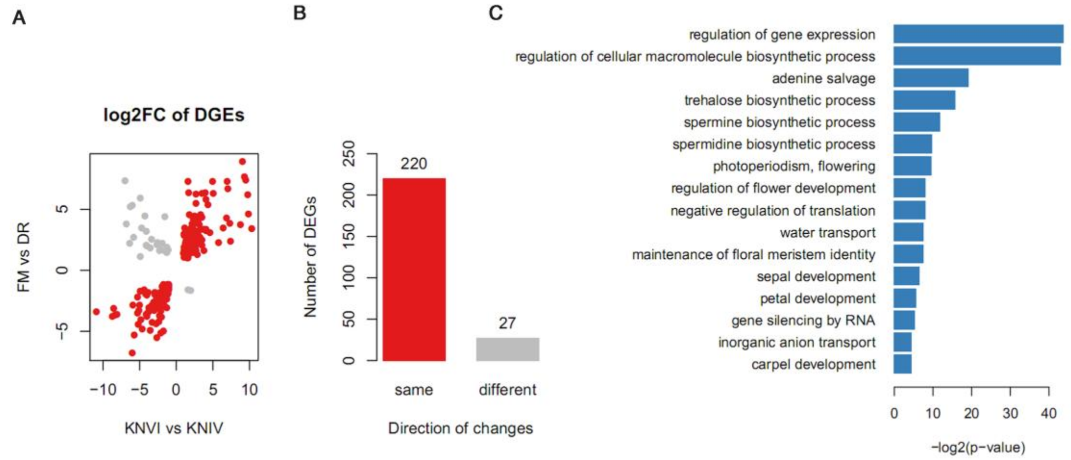

Figure S6. Comparison of differentially expressed genes (DEGs) between double ridge stage and floret differentiation stage in our and Feng's study. (A) Scatter plot of expression level fold change between floret differentiation stage (KNVI) and double ridge stage (KNIV) in our study (KNVI vs KNIV) and Feng's paper (FM vs DR). Red and grey dots indicate positive and negative correlation, respectively. (B) Number of DEGs having the same or different direction of changes. (C) Go terms that are enriched in the DEGs with the same direction of changes.

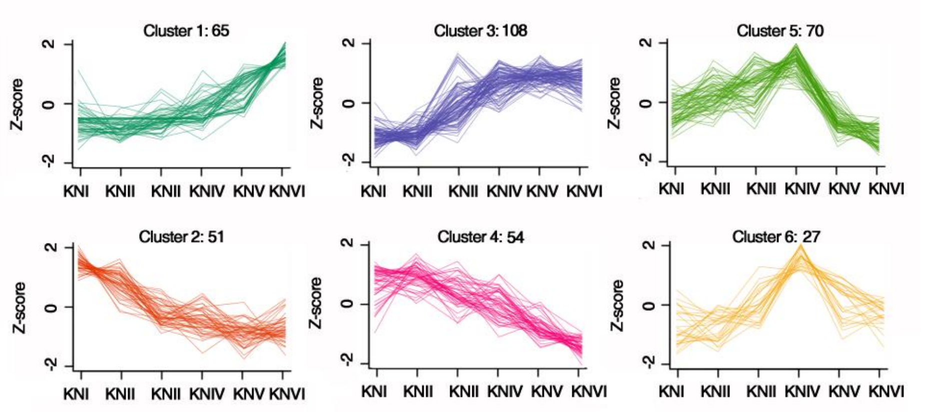

Figure S7. Expression trend of transcription factor (TF) genes in six clusters during six developmental stages. Thin lines represent the expression levels of individual TF genes. These six clusters correspond to the clusters in Figure 3.

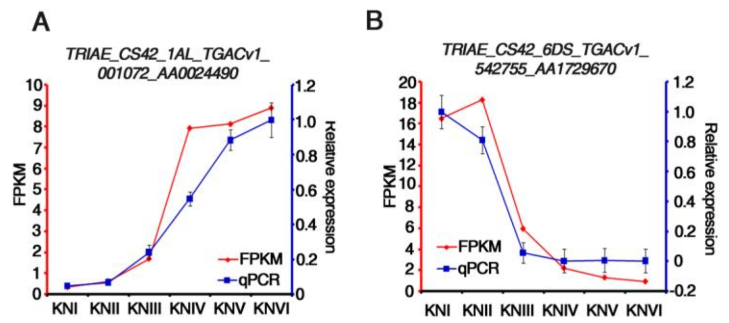

Figure S8. RT-qPCR validation of the expression patterns at six developmental stages for two *SOC1* homologous genes. (A) RT-qPCR validation of the expression patterns for *TRIAE_CS42_1AL_TGACv1_001072_AA0024490*. (B) RT-qPCR validation of the expression patterns for *TRIAE_CS42_6DS_TGACv1_542755_AA1729670*.

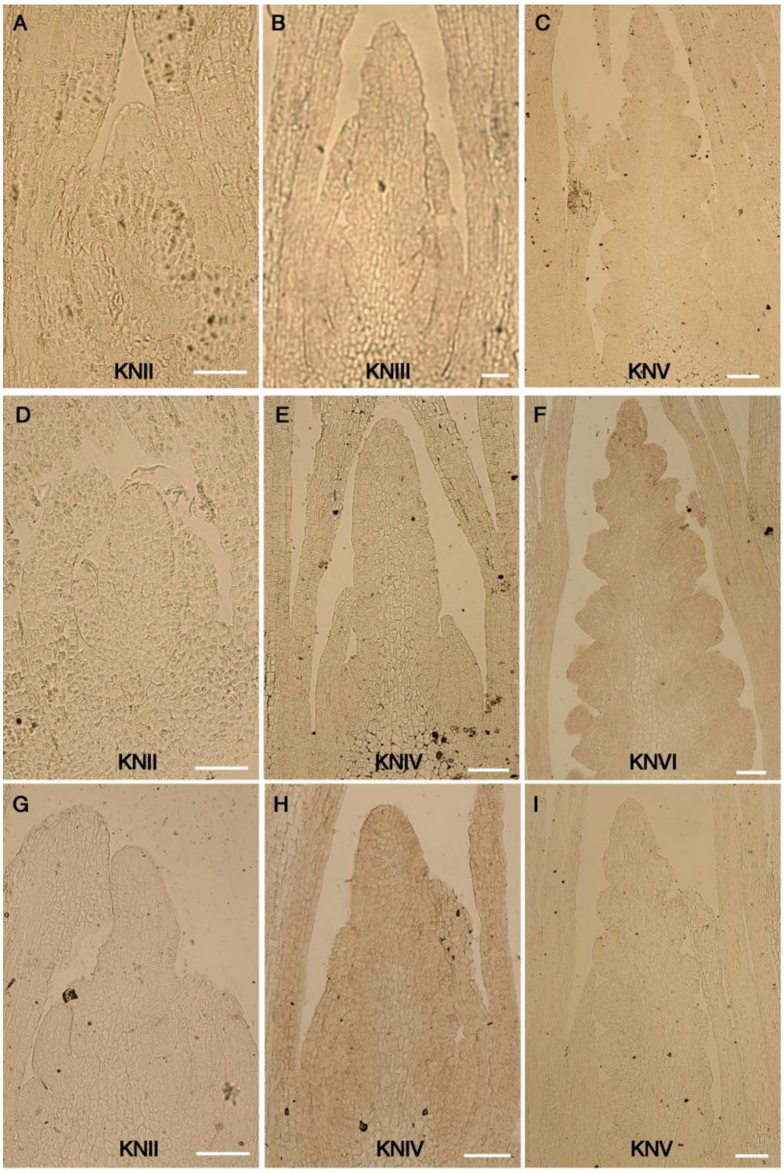

Figure S9. *In situ* hybridization of putative *TaSVP*, *TaLAX1* , and *TabHLH27* with sense probe as control. (A-C): putative *TaSVP*, (D-F): putative *TaLAX1*, (G-I): *TabHLH27*. Development stages are indicated. Bars: 100 μm.

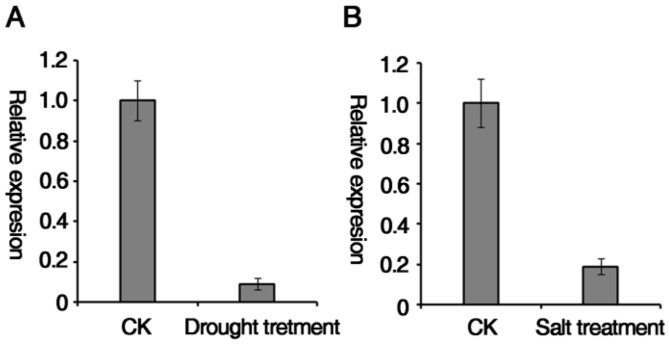

Figure S10. Histogram showing expression change of *TabHLH27* after drought (A) or salt (B) treatment. The expression were detected in young spike at KNIV stage. *TaACTIN* served as the internal control, and the expression level were relative to normal environment.

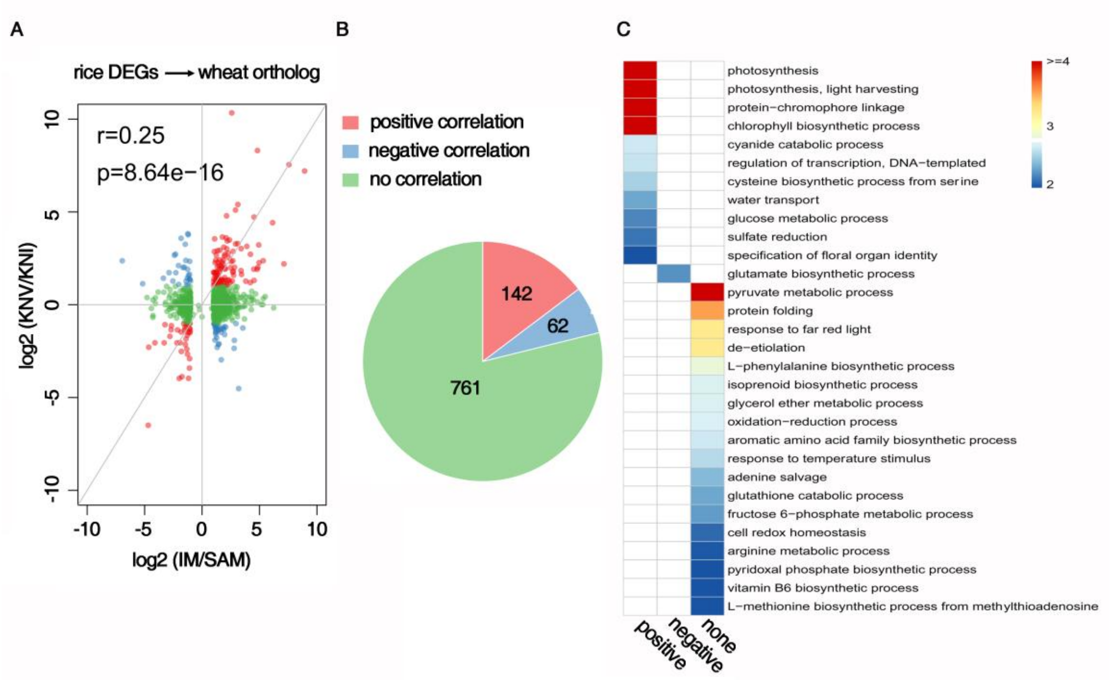

Figure S11. Comparison of differentially expressed genes (DEGs) between the inflorescence meristem (IM) and shoot apical meristem (SAM) during wheat and rice spike development. (A) Scatter plot of log2(fold change) of rice DEGs and wheat homologous genes. The average log2(fold change) values were calculated if multiple rice genes were the best homolog to a single wheat gene. Red, blue and green dots indicate positive, negative and no correlation, respectively. r, Pearson correlation coefficient; p, p-value. (B) Number of rice and wheat homologous genes showing positive (red), negative (blue) or no (green) correlation in expression fold change. (C) GO terms that were enriched among wheat homologous genes with positive, negative and no correlation. Each square is colored according to the –log10(p) value, where p is the p-value for the significance of GO term enrichment.
